# Dickkopf-1 expression is associated with tumorigenity and lymphatic metastasis in human hilar cholangiocarcinoma

**DOI:** 10.18632/oncotarget.11859

**Published:** 2016-09-06

**Authors:** Xiang-de Shi, Xian-huan Yu, Wen-rui Wu, Xiao-lin Xu, Jie-Yu Wang, Lei-bo Xu, Rui Zhang, Chao Liu

**Affiliations:** ^1^ Guangdong Provincial Key Laboratory of Malignant Tumor Epigenetics and Gene Regulation and Department of Biliary-Pancreatic Surgery, Sun Yat-sen Memorial Hospital, Sun Yat-sen University, Guangzhou, 510120, China; ^2^ Department of Ultrasound, Sun Yat-sen Memorial Hospital, Sun Yat-sen University, Guangzhou, 510120, China; ^3^ Department of Hematology, Sun Yat-sen Memorial Hospital, Sun Yat-sen University, Guangzhou, 510120, China; ^4^ Faculty of Medicine, Department of Gastroenterology and Hepatology, University Duisburg-Essen, Essen, 45147, Germany

**Keywords:** DKK1, HCCA, tumorigenesis, lymphatic metastasis

## Abstract

Dickkopf-1 (DKK1) is involved in tumorigenesis and the invasion of several tumors. However, its biological function in human hilar cholangiocarcinoma (HCCA) has not yet been documented. This study was designed to investigate the clinical significance and biological function of DKK1 in HCCA. The expression of DKK1 was investigated in thirty-seven human HCCA biopsy samples by immunohistochemistry. To further explore the biological effects of DKK1 in HCCA, transient and stable knockdown of DKK1 in two human HCCA cells (QBC939 and FRH0201) were established using small interfering or short hairpin RNA expression vector. In the present study, immunohistochemistry revealed that DKK1 was up-regulated in human HCCA tissues (24/37, 64.9%). High levels of DKK1 in human HCCA correlated with metastasis to the hilar lymph nodes (P=0.038). Genetic depletion of DKK1 in HCCA cells resulted in significantly inhibited proliferation, colony formation and migration compared with controls. Most importantly, DKK1 down-regulation impaired tumor formation capacity of HCCA cells *in vivo*. Subsequent investigations revealed that β-catenin is an important target of DKK1 and DKK1 exerts its pro-invasion function at least in part through the β-catenin/ matrix metalloproteinase-7 (MMP-7) signaling pathway. Consistently, in human HCCA tissues, DKK1 level was positively correlated with β-catenin and MMP-7 expression, as well as tumor hilar lymphatic metastasis. Taken together, our findings indicate that DKK1 may be a crucial regulator in the tumorigenicity and invasion of human HCCA, DKK1 exerts its pro-invasion function at least in part through the β-catenin/ MMP-7 signaling pathway, suggesting DKK1 as a potential therapeutic target for HCCA.

## INTRODUCTION

Hilar cholangiocarcinoma (HCCA) is the second most common primary liver cancer, which originates from the hilar biliary duct epithelium and is a highly invasive cancer accounting for 50 to 60% of cholangiocarcinoma [1, 2]. Epidemiologic studies indicate an increasing incidence of HCCA, which has become one of the major malignancies threatening human health in recent years [3]. Radical surgical resection is the most effective therapy for the treatment of HCCA [4]. Due to frequent local recurrence of the tumor, the postoperative 5-year survival rate is <30%, even in patients who undergo radical surgery [5, 6]. For inoperable patients, the median survival time is 6 to 12 months and the overall 5-year survival rate is <5% [7, 8]. Regional and para-aortic lymph nodes are frequently involved in HCCA, and lymph node metastasis (LNM) remains one of its most important prognostic markers. Thus, biomarkers that can predict the risk of recurrence and metastasis are urgently needed. It is necessary to identify novel gene targets that participate in tumor progression and to design appropriate treatment strategies for HCCA patients.

The Dickkopf (DKK) family consists of four members (DKK1, DKK2, DKK3 and DKK4). DKK1 is the most studied member of the DKK family. DKK1 was originally identified as a head inducer after its mRNA was injected into Xenopus embryos [9]. In the canonical Wnt/β-catenin pathway, Wnt1 protein binds to the frizzled receptor (Fz) and the low-density lipoprotein receptor-related protein [10, 11]. It has been reported that the expression of DKK1 were downregulated, resulted in β-catenin degradation and retardation of proliferation of tumor cells [12, 13]. However, recent studies have reported that DKK1 is up-regulated in many tumors, including breast cancer, lung cancer, esophageal carcinoma and hepatocellular carcinoma (HCC) [14–18]. Moverover, DKK1 is involved in the invasion of non-small cell lung cancer, pancreatic cancer and HCC [19–21]. Shi et al. demonstrated that high expression of DKK1 is related to lymphatic metastasis and is indicative of a poor prognosis in intrahepatic cholangiocarcinoma (ICC) patients after surgery. Conversely, depletion of DKK1 using small interfering RNA results in a decrease in ICC cell migration and invasion [22]. Taken together, these findings suggest that DKK1 performs an oncogenic and invasion function. However, whether DKK1 acts as an oncogene in HCCA remains unclear. Furthermore, the precise mechanism of DKK1 involvement in HCCA tumorigenesis and invasion has not been determined. This study was designed to investigate the clinical significance and biological function of DKK1 in human HCCA.

## RESULTS

### The expression of DKK1 was elevated in HCCA tissues and associated with hepatic hilar lymph nodes metastasis

**Figure 1:**
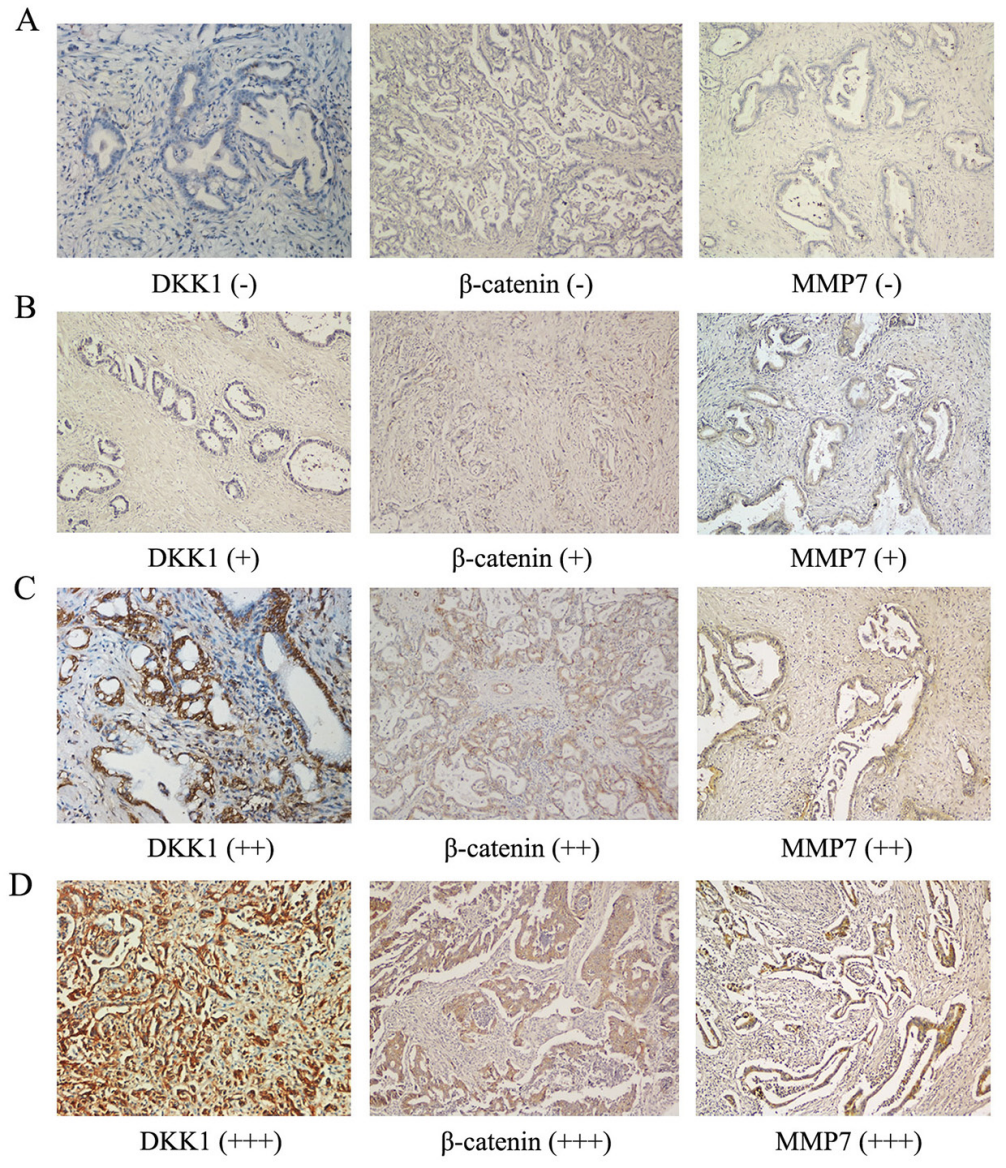
Expression of DKK1, β-catenin and MMP-7 in human HCCA tissues Immunohistochemistry revealed no staining (−) **A.** low staining (+) **B.** moderate staining (++) **C.** and strong staining (+++) **D.** for DKK1, β-catenin and MMP-7 in human HCCA tissue. The DKK1 level was positively correlated with β-catenin and MMP-7 expression in human HCCA tissue (A-D). *P<0.05.

Positive staining of DKK1 protein by immunohistochemistry was observed in the cytoplasm of tumor cells (Figure 1). The expression of DKK1 was up-regulated in HCCA tissues compared with corresponding peritumoral tissues (Supplementary Figure S1A-S1B). In general, high DKK1 expression (++ or +++) was observed in 24 of 37 tumor samples (64.9%; Figure 1C-1D), whereas low DKK1 expression (− or +) was noted in 13 of 37 tumor samples (35.1%; Figure 1A-1B).

As shown in Supplementary Table S1, the level of DKK1 expression in HCCA tissues was significantly correlated with metastasis to hepatic hilar lymph nodes (P=0.038) and tumor differentiation (P=0.039). No statistically significant differences were noted in gender (P=0.489), nerve infiltration (P=0.495), vascular invasion (P=0.091), TNM stage (P=0.691) or serum CA19-9 (P=0.383).

### Transfection of DKK1-shRNA repressed the proliferation and colony formation capacity of QBC939 and FRH0201 cells *in vitro*

**Figure 2:**
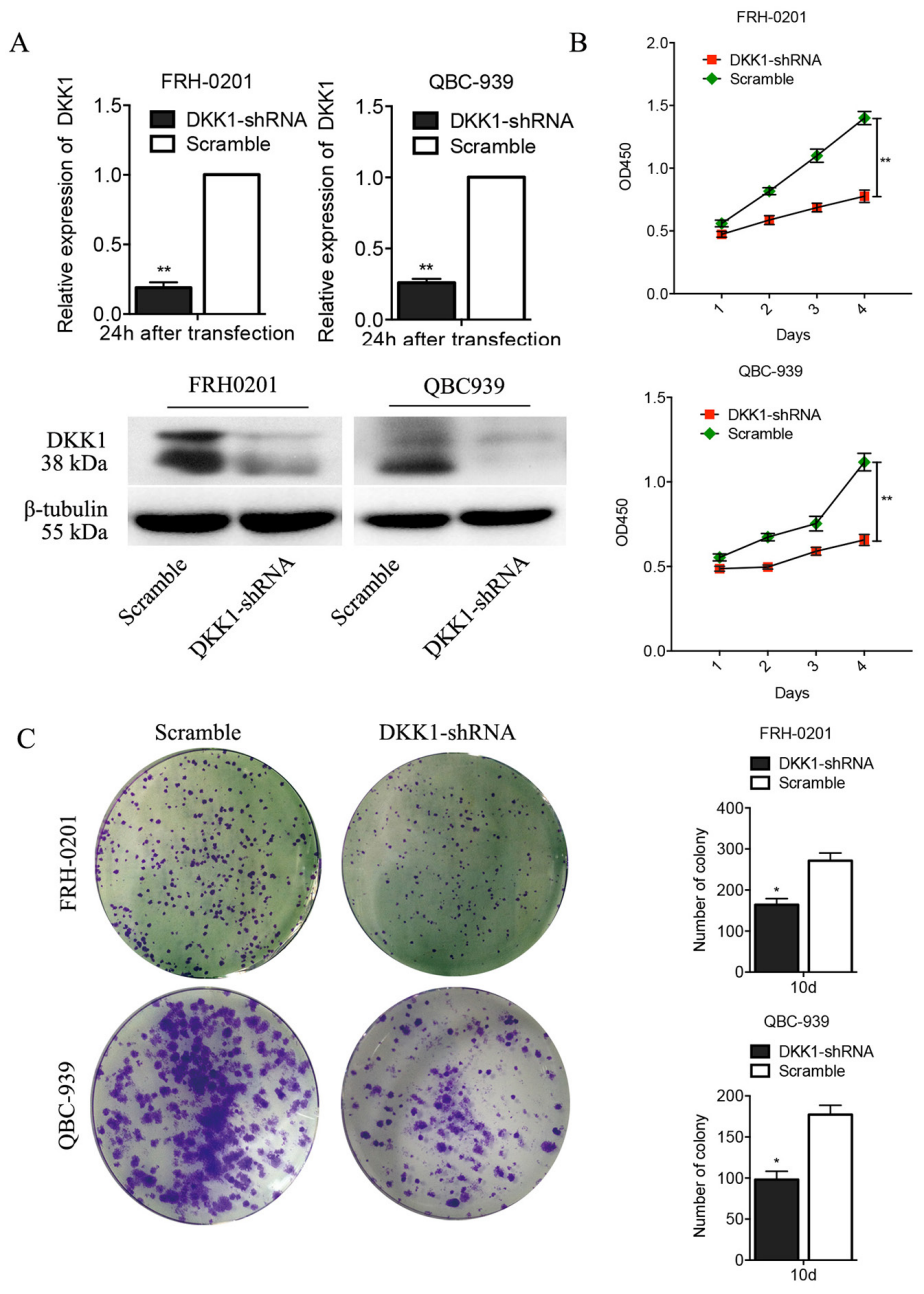
Transfection of DKK1-shRNA represses proliferation and colony formation of QBC939 and FRH0201 cells *in vitro* Real time PCR analysis revealed that DKK1 mRNA in DKK1-shRNA QBC939 and FRH0201 cells was significantly down-regulated **A.** Similar results were obtained by Western blotting analysis (A). Down-regulation of DKK1 expression by DKK1-shRNA significantly inhibited the proliferation of QBC939 and FRH0201 cells **B.** The number of colony was lower in DKK1-shRNA QBC939 and FRH0201 cells group than the control group(C). *P<0.05.

To investigate the effect of DKK1 on HCCA cells, we depleted its expression in QBC939 and FRH0201 cells by shRNA-DKK1 and established stable cell lines. As shown in Figure 2, the qRT-PCR and Western blot results revealed that DKK1 is effectively and functionally suppressed in QBC939 and FRH0201 cells compared to the scramble control (Figure 2A). The results of western blot were quantified by Image J (Supplementary Figure S1C). To further explore the biological role of reduced DKK1 in of QBC939 and FRH0201 cells, proliferation assay and colony formation assays were performed. CCK-8 cell proliferation assay revealed that the decrease in DKK1 expression caused by DKK1-shRNA significantly inhibited the proliferation of QBC939 and FRH0201 cells (Figure 2B). A colony formation assay, based on crystal violet staining, revealed that fewer colonies were found in the DKK1-shRNA-treated QBC939 and FRH0201 cells compared to the control(Figure 2C). Similar results were observed in QBC939 and FRH0201 cells tranfected with small interfering (si) RNA (Supplementary Figure S2A-S2D). Collectively, these data indicate that down-regulation of DKK1 inhibits the proliferation and colony formation capacity of QBC939 and FRH0201 cells.

### Transfection of DKK1-shRNA impaired the migration of QBC939 and FRH0201 cells *in vitro*

**Figure 3:**
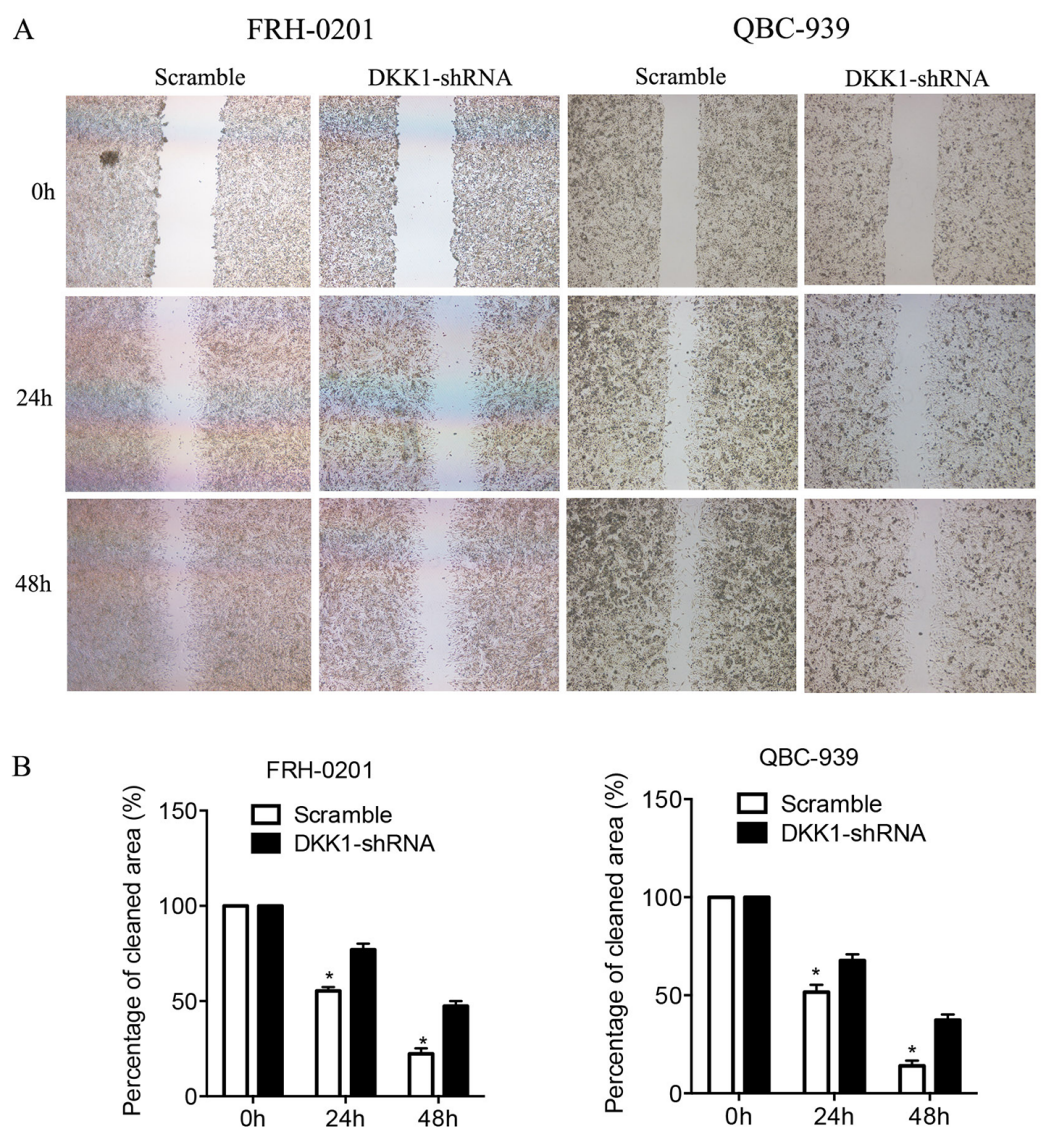
Transfection of DKK1-shRNA represses the migration of QBC939 and FRH0201 cells *in vitro* In a wound healing assay, QBC939 and FRH0201 cells in the DKK1-shRNA group exhibited decreased migration ability compared with NC-shRNA group **A.** Cells were monitored every 24 h for 2 days to evaluate the rate of migration into the scratched area **B.** *P<0.05.

To examine whether the targeted down-regulation of DKK1 in QBC939 and FRH0201 cells affects the migration of tumor cells, *in vitro* wound healing assays were performed. We found that cells in the DKK1-shRNA group exhibited decreased migration ability compared with the control (Figure 3A-3B). Similar results were observed in QBC939 and FRH0201 cells tranfected with siRNA (Supplementary Figure S2E). Thus, down-regulation of DKK1 dramatically diminishes the migration of QBC939 and FRH0201 cells *in vitro*.

### Transfection of DKK1-shRNA impaired tumor formation of QBC939 and FRH0201 cells *in vivo*

**Figure 4:**
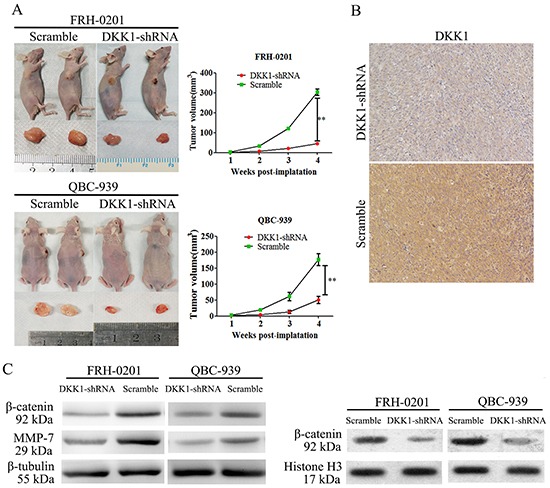
Transfection of DKK1-shRNA impaired QBC939 and FRH0201 cell tumor formation *in vivo* Following down-regulation of DKK1 expression, QBC939 and FRH0201 cells exhibited significantly diminished in vivo tumor formation ability compared with control cells. Control group QBC939 and FRH0201 cells generated large tumors on the left flanks of nude mice **A, left panel.** whereas smaller tumors were observed on the right flanks injected with DKK1-shRNA QBC939 and FRH0201 cells **A, left panel.** The tumor volume of the recipient mice is presented **A, right panel.** The expression of DKK1 was positively correlated with subcutaneous tumor volume **B.** Western blot revealed that shRNA-DKK1 remarkably decreased the levels of β-catenin **C.** MMP7(C) and nuclear β-catenin **D.** protein levels of QBC939 and FRH0201 cells compared with control cells. *P<0.05.

Because our *in vitro* studies suggested that DKK1 plays a regulatory role in QBC939 and FRH0201 cell proliferation and migration, the biological significance of these results was further evaluated in an in vivo model of HCCA. To this end, both DKK1-shRNA tumor cells and control cells were implanted subcutaneously into nude mice, and the resulting tumor was measured. Following down-regulation of DKK1 expression, QBC939 and FRH0201 cells exhibited significantly diminished *in vivo* tumor formation compared with control cells (Figure 4A). DKK1 expression in subcutaneous tumors was analyzed by immunohistochemistry. DKK1 expression was positively correlated with subcutaneous tumor volume, the representative images are shown in Figure 4B. These data corroborate our *in vitro* observations and support the notion that DKK1 may play a vital role on proliferation and tumorigenicity of HCCA cells.

### Knockdown of DKK1 repressed β-catenin expression in QBC939 and FRH0201 cells

We next investigated a potential mechanism for DKK1-mediated HCCA cell migration and invasion. DKK1 is a key regulator of the Wnt/β-catenin signaling pathway. We therefore examined whether DKK1 influenced β-catenin expression in HCCA. First, we analyzed β-catenin expression using immunohistochemistry in tissue from all 37 cases of HCCA. Interestingly, DKK1 levels were positively correlated with β-catenin expression (Figure 1A-1D and Table 1; P=0.021). Consistently, Western blotting indicated that DKK1-shRNA inhibited β-catenin expression in QBC939 and FRH0201 cells compared with control cells (Figure 4C). Most importantly, the expression of nuclear β-catenin was impaired by DKK1-shRNA in QBC939 and FRH0201 cells (Figure 4D). The results of western blot were quantified by Image J (Supplementary Figure S1D-S1F). These findings indicate that β-catenin may be an important downstream target of DKK1.

**Table 1:** Correlation between DKK1 expression and 37 patients with hilar cholangiocarcinoma

Variables		DKK1		
		Positive	Negative	P
Sex	Male	15	6	0.489
	Female	9	7	
CA19-9(U/L)	≥37	18	12	0.383
	<37	6	1	
Tumor differentiation	Well	6	8	0.039
	Poor	18	5	
Nerve infiltration	Yes	8	6	0.495
	No	16	7	
Hilar lymphatic metastasis	Yes	15	3	0.038
	No	9	10	
Vascular invasion	Yes	15	4	0.091
	No	9	9	
TNM stage	I-II	5	4	0.691
	III-IV	19	9	
Beta-catenin	Positive	16	4	0.021
	Negative	7	10	
MMP-7	Positive	18	5	0.039
	Negative	6	8	

### Matrix metalloproteinase-7 (MMP-7) was an important downstream target of the DKK1/β-catenin signaling pathway

MMP-7 is one of the most important target genes downstream of β-catenin signaling pathway and MMP-7 plays a crucial role in promoting cancer migration and invasion [23]. We therefore investigated whether DKK1 exerted its pro-invasion function through MMP-7 in HCCA. We analyzed MMP-7 expression using immunohistochemistry on tissues from all 37 cases of human HCCA. MMP-7 levels were positively correlated with DKK1 and β-catenin expression (Figure 1A-1D and Table 1; P=0.039). Most importantly, the expression of DKK1, β-catenin and MMP-7 in HCCA were associated with hilar lymph nodes metastasis. Consistently, as shown in Figure 4C, the introduction of shRNA-DKK1 remarkably decreased MMP-7 protein levels in QBC939 and FRH0201 cells. The results of western blot were quantified by Image J (Supplementary Figure S1D-S1F). Taken together, our findings suggest that DKK1 promotes HCCA cells migration and invasion, at least in part through promoting β-catenin/MMP-7 signaling.

## DISCUSSION

DKK1 functions as key regulator of the canonical Wnt pathway [24]. DKK1 binds to LRP5/6 and dysregulation its interaction with Wnt, resulting in β-catenin degradation [10]. Numerous studies have demonstrated that DKK1 is involved in tumorigenesis in many types of cancer, including breast cancer, lung cancer, esophageal carcinomas, HCC and malignant bone disease [15, 25, 26, 27]. However, the role of DKK1 in the progress of HCCA has not been documented. In the present study, we found that the expression of DKK1 is elevated in HCCA tissues, and the high level of DKK1 expression was correlated with hilar lymph node metastasis. These data indicate that DKK1 may involve in the lymph node metastasis of HCCA.

Studies have indicated that DKK1 is oncogenic and is involved in invasive growth in non-small cell lung cancer cells [19]. Chen et al. reported that DKK1 promotes the invasion and metastasis of HCC cells [21]. A recent research article demonstrated that high DKK1 expression is related to lymphatic metastasis and indicates poor prognosis in ICC [22]. However, the role of DKK1 in the invasion and tumorigenicity of HCCA cells is still unclear. Herein, our *in vitro* assays demonstrated that the depletion of DKK1 in these cells impaired proliferation, colony formation capacity and migration capacity. Most importantly, implantation of DKK1-shRNA HCCA cells into nude mice impaired their tumor formation capacity *in vivo*. Immuno-histochemistry revealed that DKK1 expression was positively correlated with subcutaneous tumor volume. These data corroborate our *in vitro* observations and support the notion that DKK1 may play a vital role on progression of HCCA.

We therefore studied the mechanism of DKK1 in the tumorigenesis and invasion of HCCA. DKK1 is a key regulator of Wnt/β-catenin signaling pathway. The Wnt/β-catenin signaling pathway is highly conserved in evolutionary processes and is up-regulated in multiple tumors [28]. However, the role of DKK1 on the expression of β-catenin is controversial [29]. Although DKK1's ability to inhibit the canonical Wnt/β-catenin signalling pathway have been identified first, it was later found that the DKK1 gene is also a target of Wnt/β-catenin activation. It has been reported that DKK1 could down-regulate the expression of β-catenin in breast cancer, thyroid cancer and epidermal neoplasms [30–32]. In contrast, Chen et al. reported that up-regulation of DKK1 expression noticeably promoted the cytoplasmic and nuclear accumulation of β-catenin in HCC cells [25]. In fact, the DKK1 promoter region contains several putative T-cell factor (TCF)-binding sites and was shown to be a direct target of activated β-catenin [33]. In addition, secreted DKK1 could block its own transcription, thus creating a negative feedback loop [34]. In the present study, immunohistochemistry revealed that DKK1 levels were positively correlated with β-catenin expression in tissue samples from all thirty-seven HCCA cases. In addition, the expression of β-catenin and nuclear β-catenin were dramatically decreased in DKK1-shRNA QBC939 and FRH0201 cells. These findings indicate that β-catenin may be an important target downstream of DKK1.

MMP-7, also known as matrilysin, is a secreted protein implicated in the destruction of a broad range of extracellular matrix substrates in various cancers [35, 36]. MMP-7 is one of the most important target genes downstream of β-catenin signaling and plays a crucial role in promoting tumor cell migration and invasion [37, 38]. Recently, several reports have shown that MMP-7 is up-regulated in both cholangiocarcinoma cells and tissues [39–41]. DKK1 promotes HCC cell migration and invasion through MMP-7 [21]. However, the relationship between DKK1 and MMP-7 in HCCA remains unclear. Herein, we demonstrate that MMP-7 is an important target downstream of DKK1 in HCCA cells. DKK1-shRNA markedly decreased β-catenin and MMP-7 protein levels in QBC939 and FRH0201 cells. Consistently, in human HCCA tissues, DKK1 level was positively correlated with β-catenin and MMP-7 expression, as well as tumor hilar lymphatic metastasis. Our findings suggest that DKK1 promotes HCCA cell migration and invasion, at least in part through promoting β-catenin/MMP-7 signaling.

In conclusion, our results indicate that DKK1 may be a crucial regulator in the progression of human HCCA, DKK1 exerts its pro-invasion function at least in part through the β-catenin/ MMP-7 signaling pathway, suggesting that DKK1 may be a potential target for HCCA therapy.

## MATERIALS AND METHODS

### Patients and tissue specimens

Tumor specimens were obtained from thirty-seven consecutive HCCA patients who underwent resection at the Department of Biliary-Pancreatic Surgery, Sun Yat-sen Memorial Hospital. None of the patients received chemotherapy or radiation therapy prior to radical tumor resection. The difference and significance of DKK1 expression among those patients were investigated. The detailed clinicopathological characteristics of all patients are presented in Table 1. The project was approved by the ethics committee of the Sun Yat-sen Memorial Hospital. Written informed consent was obtained from either the patient or guardian.

### Immunohistochemistry

Immunohistochemistry was performed according to the protocol provided with the PV Two-Step Kit instructions and as previously described [42]. Details regarding primary and secondary antibodies are provided in Supplementary Table S1. DKK1, β-catenin and MMP-7 expression was evaluated under a light microscope. The staining was independently evaluated by two investigators who were unaware of the clinical data. For each specimen, five images of representative areas were acquired, and tumor cells were counted. The percentages of positively stained cells were determined by examination under a microscope of 30 randomly selected foci, which were each composed of more than 100 cells. The intensity of DKK1 staining was evaluated as follows: strongly positive samples (scored as +++) had dark brown staining in > 50% of tumor cells, completely obscuring the cytoplasm; moderately positive (scored as ++) had dark brown staining in 25 to 50% of tumor cells obscuring the cytoplasm; weakly positive (+) showed a lesser degree of brown staining in the tumor cell cytoplasm; and “absent” (scored as -) exhibited no appreciable staining in tumor cells. Strong and moderate scores were regarded as positive results [22, 25].

### Cell culture

Two human HCCA cell lines QBC939 and FRH0201 were obtained from the Cell Bank of the Chinese Academy of Sciences (Shanghai, China) and cultured in RPMI 1640 (Invitrogen Co., Carlsbad, CA) supplemented with 10% heat-inactivated fetal bovine serum (Invitrogen) as recommended by the supplier. All cultures were maintained in a humidified atmosphere containing 5% CO_2_ at 37°C.

### Virus production and transduction

Lentiviral vectors expressing short hairpin RNA (shRNA) targeting human DKK1 (target sequence: sh-DKK1-1, 5-GATCCGTACCAAGCATAGGAGAAATTCAAGAGATTTCTCCTATGCTTGGTACTTTTTTG-3; sh-DKK1-2, 5-AATTCAAAAAAGTACCAAGCATAGGAGAAATCTCTTGAATTTCTCCTATGCTTGGTACG-3)and a negative control were purchased from GenePharma (Shanghai, China). All shRNA sequences were subjected to basic local alignment search tool (BLAST) search to confirm the absence of homology to any additional known coding sequences. QBC939 and FRH0201 cells were transfected with shRNA-DKK1. After puromycin selection, loss of DKK1 was confirmed by Quantitative RT-PCR (qRT-PCR) and Western blot analysis, and stable cell lines were established. A validated siRNA targeting DKK1 and the negative control were purchased from GenePharma (Shanghai, China). Transfection was performed as described previously [43].

### Quantitative RT-PCR and western blot analyses

Knockdown of DKK1 in QBC939 and FRH0201cells was confirmed by real time polymerase chain reaction (qRT-PCR) as described [42]. Primer sets used for qRT-PCR amplification are presented in Supplementary Table S2. Western blot analyses were performed as described [42], protein lysates obtained from the cultured cells were subjected to sodiumdodecylsulfate-polyacrylamidegelelectrophoresis (SDS-PAGE) and were probed with primary antibodies recognizing DKK1 (1:2000), β-catenin (1:5000) and MMP-7(1:5000). After incubation with appropriate horseradish peroxidase (HRP)-conjugated secondary antibodies (Jackson Innumoresearch, USA), protein bands were visualized using enhanced chemiluminescence (ECL) plus Western blotting detection reagents followed by exposure to Hyper-films (Amersham, UK). Details regarding the primary and secondary antibodies were provided in Supplementary Table S1.

### Cell proliferation assay

Cell proliferation assay was performed as previously described [42]. Briefly, HCCA cells were inoculated in 96-well plates at the density of 5 × 10^3^/well for 24 h, and 3 repetitive wells were prepared for each group. After the corresponding treatments, the cells were treated with 10 μl Cell Counting Kit-8 (CCK-8) (Dojindo Molecular Technologies) in an incubator for 2h. The absorbance at 450 nm was measured by an enzyme linked immunosorbent assay (ELISA) plate reader to calculate the cell growth inhibition rate (%) according to the formulation: Inhibition rate (%) = (OD_blank_ - OD_experiment_)/OD_blank_ ×100%. The absorbance of the negative control (OD) was considered to be 0%.

### Colony formation assay

HCCA cells were plated in triplicate in six-well plates. After 7 days, the cells were rinsed with phosphate-buffered saline (PBS) twice, fixed with 10% formaldehyde, and stained with 0.1% crystal violet in 10% ethanol and the numbers of colonies were counted.

### Wound healing assay

Briefly, 8 × 10^5^ HCCA cells were cultured on a 35-mm dish and incubated overnight. A wound was created by scratching with a 250-μl microtip, and the cells were washed once with complete medium to remove detached cells. An image of the original wound was captured under a microscope. Dishes were then returned to the incubator for 24 h, and a second image of the wound was obtained. The width of the wound was measured with AxioVision LE software (Zeiss, Oberkochen, Germany). The difference between the widths is taken as the migration distance.

### In vivo subcutaneous xenografts

All animal experimentation described in this study was performed in accordance with protocols approved by the Institutional Animal Care and Use Committee at Sun Yat-sen University. Briefly, 5 × 10^6^ shRNA-DKK1 HCCA cells and control cells were suspended in 100 μl PBS and were injected subcutaneously into six female nude mice (Balb/c nu/nu) (3-4 weeks old), respectively. Tumor volumes were monitored every 7 days by measuring the length and width with a caliper and using the formula (width^2^) × length/2. Mice were sacrificed 8 weeks after injection, and the tumors were isolated and measured.

### Statistics

All experiments were performed at least in triplicate. Statistical analysis was conducted with the SPSS software package (version 13.0; SPSS, Chicago, IL). All experiments for cell cultures were carried out independently at least three times and in triplicate each time. All data were presented as mean ± standard deviation (SD), we determined the significance of differences in the human HCCA data using Fisher's exact test, in the in vitro data using Student's t test, and in the in vivo data using the Mann-Whitney U test. For all tests, a p-value less than 0.05 is considered to be statistically significant and indicated by asterisks in the figures. All p-values reported are the result of two-sided tests.

## SUPPLEMENTARY FIGURES AND TABLES


