# Supplementary material for: Dickkopf-1 expression is associated with tumorigenity and lymphatic metastasis in human hilar cholangiocarcinoma
Source: Oncotarget. 2016 Sep 6;7(43):70378–87. doi: 10.18632/oncotarget.11859 (PMC5342559; doi:10.18632/oncotarget.11859)
Supplement: Supplementary file 1 [file oncotarget-07-70378-s001.pdf]

Dickkopf-1 expression is associated with tumorigenity and lymphatic metastasis in human hilar cholangiocarcinoma

SUPPLEMENTARY FIGURES AND TABLES

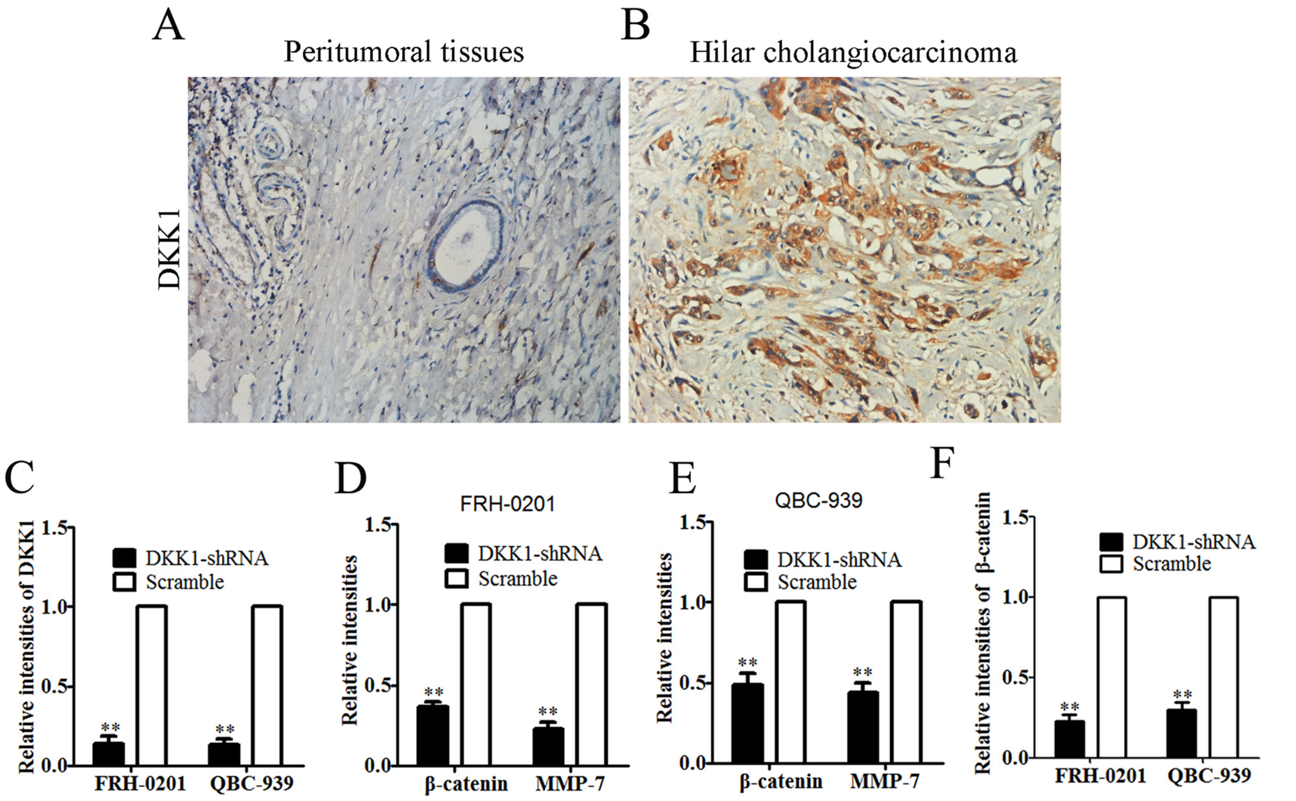

Supplementary Figure S1: Expression of DKK1 in HCCA tissues and corresponding peritumoral tissues. The expression of DKK1 was up-regulated in HCCA tissues compared with corresponding peritumoral tissues (Supplementary Figure S1A-S1B). The protein levels of DKK1 **C.** β-catenin **D-E.** MMP-7 (D-E) and nuclear β-catenin **F.** in QBC939 and FRH0201 were quantified by ImageJ . *P<0.05, **P<0.01.

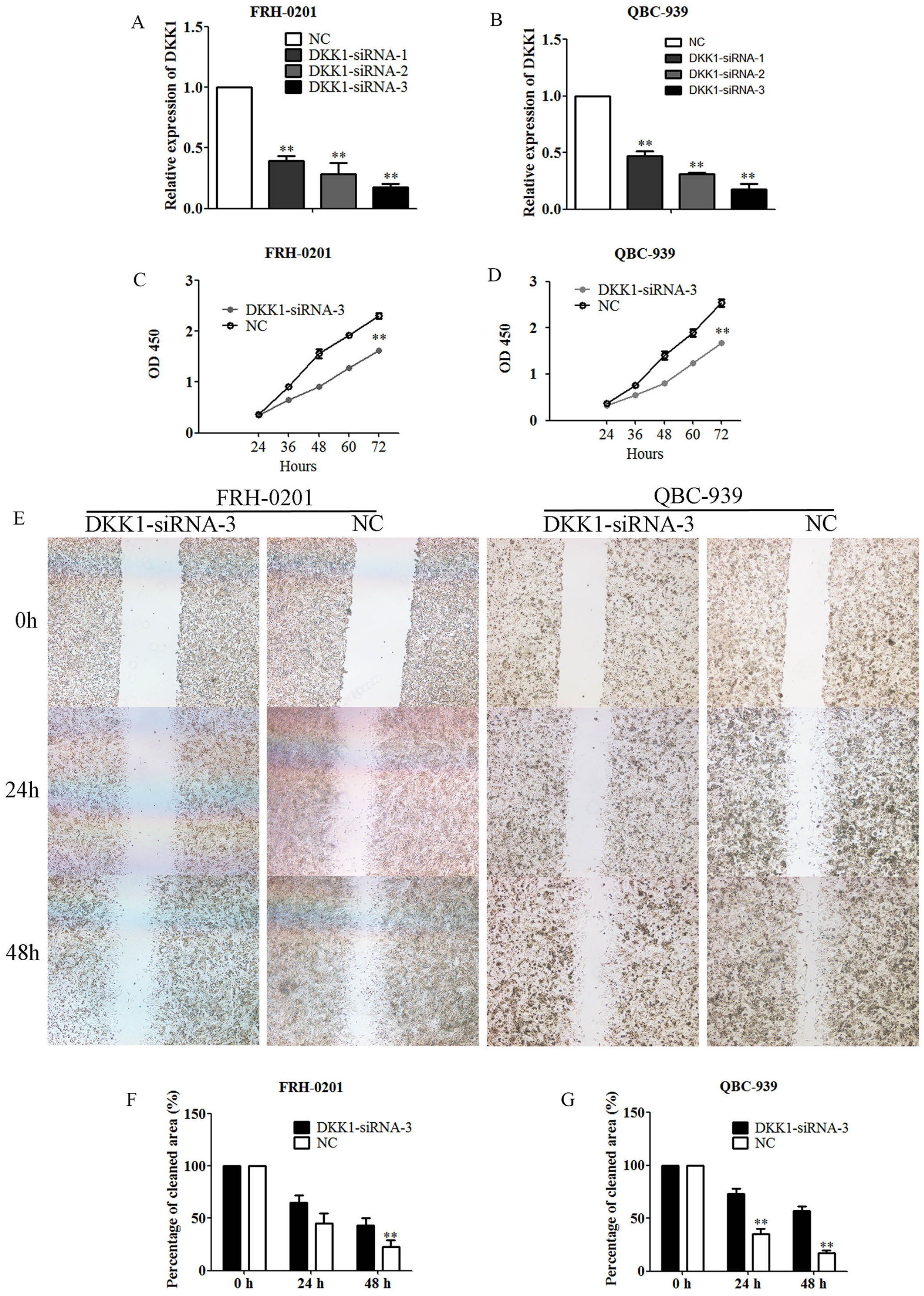

Supplementary Figure S2: Transfection of DKK1-siRNA represses proliferation and migration of QBC939 and FRH0201 cells *in vitro*. Real time PCR analysis revealed that DKK1 mRNA in DKK1-shRNA QBC939 and FRH0201 cells was significantly down-regulated **A-B.** Down-regulation of DKK1 expression by DKK1-siRNA significantly inhibited the proliferation of QBC939 and FRH0201 cells **C-D.** In a wound healing assay, QBC939 and FRH0201 cells in the DKK1-siRNA group exhibited decreased migration ability compared with NC-shRNA group **E.** Cells were monitored every 24 h for 2 days to evaluate the rate of migration into the scratched area **F-G.** **P<0.01.

Supplementary Table S1: The information of antibodies used in this study

Antibodies	Company	Product Number	Dilution
DKK1	Abcam	ab109416	1:300 (IHC) 1:2000 (WB)
β-catenin	Abcam	ab32572	1:500 (IHC) 1:5000 (WB)
Histone H3	Abcam	ab1791	1:2000 (WB)
β-tubulin	Abcam	ab179513	1:1000 (WB)
MMP-7	Abcam	ab205525	1:1500 (IHC) 1:1000 (WB)
HRP-linked anti-rabbit IgG	Cell Signaling Technology	#7074	1:2000 (WB)

Supplementary Table S2: Primers for DKK1 and reference genes

Gene	Primer	Sequence
DKK1	Forward	5'- GACCCAGGCTTGCAAAGTGAC-3'
	Reverse	5'-GCCCAGAGCCATCATCTCAG-3'
GAPDH	Forward	5'-GCACCGTCAAGGCTGAGAAC-3'
	Reverse	5'-TGGTGAAGACGCCAGTGGA-3'
